# TORC1 Promotes Phosphorylation of Ribosomal Protein S6 via the AGC Kinase Ypk3 in *Saccharomyces cerevisiae*

**DOI:** 10.1371/journal.pone.0120250

**Published:** 2015-03-13

**Authors:** Asier González, Mitsugu Shimobayashi, Tobias Eisenberg, David Adrian Merle, Tobias Pendl, Michael N. Hall, Tarek Moustafa

**Affiliations:** 1 Biozentrum, University of Basel, Basel, Switzerland; 2 Institute of Molecular Biosciences, University of Graz, Graz, Austria; Florida State University, UNITED STATES

## Abstract

The target of rapamycin complex 1 (TORC1) is an evolutionarily conserved sensor of nutrient availability. Genetic and pharmacological studies in the yeast *Saccharomyces cerevisiae* have provided mechanistic insights on the regulation of TORC1 signaling in response to nutrients. Using a highly specific antibody that recognizes phosphorylation of the *bona fide* TORC1 target ribosomal protein S6 (Rps6) in yeast, we found that nutrients rapidly induce Rps6 phosphorylation in a TORC1-dependent manner. Moreover, we demonstrate that Ypk3, an AGC kinase which exhibits high homology to human S6 kinase (S6K), is required for the phosphorylation of Rps6 *in vivo*. Rps6 phosphorylation is completely abolished in cells lacking Ypk3 (*ypk3Δ*), whereas Sch9, previously reported to be the yeast ortholog of S6K, is dispensable for Rps6 phosphorylation. Phosphorylation-deficient mutations in regulatory motifs of Ypk3 abrogate Rps6 phosphorylation, and complementation of *ypk3Δ* cells with human S6 kinase restores Rps6 phosphorylation in a rapamycin-sensitive manner. Our findings demonstrate that Ypk3 is a critical component of the TORC1 pathway and that the use of a phospho-S6 specific antibody offers a valuable tool to identify new nutrient-dependent and rapamycin-sensitive targets *in vivo*.

## Introduction

Target of rapamycin (TOR) is a highly conserved serine/threonine kinase that is found in two structurally and functionally distinct complexes termed TOR complex 1 (TORC1) and TORC2 of which TORC1 is uniquely sensitive to rapamycin [1]. While almost all eukaryotes have a single *TOR* gene, several yeasts such as *S*. *cerevisiae* and *S*. *pombe* have two *TOR* genes. In budding yeast, both TOR1 and TOR2 can be part of TORC1, but TORC2 contains only TOR2 [2,3]. TORC1 acts as a nutritional sensor that couples nutrient availability to protein synthesis and cell growth. This signaling pathway controls the activity of a wide variety of kinases that help to maintain anabolic processes and antagonize catabolic processes such as autophagy and mRNA degradation [4,5].

In mammals, protein kinases of the AGC (protein kinase A/protein kinase G/protein kinase C) family are direct targets for mammalian TORC1 (mTORC1) and mTORC2. The ribosomal S6 kinase (S6K) is the best characterized target of mTORC1. S6K requires mTORC1-mediated phosphorylation in the hydrophobic motif (Thr389) as well as phosphorylation by phosphoinositide-dependent kinase 1 (PDK1) in its activation loop to be fully active [6]. Mammalian S6K phosphorylates 40S ribosomal protein S6 (S6) at five Ser residues (Ser235/236/240/244/247) to promote transcription of genes required for ribosome biogenesis [7]. Thus, S6 phosphorylation is one of the most sensitive readouts of mTORC1-dependent signaling [8].

In *S*. *cerevisiae*, the AGC kinases Sch9 and Ypk1/2 are direct targets for TORC1 and TORC2, respectively. Sch9 is considered to be the functional ortholog of mammalian S6K [9]. Budding yeast S6 (Rps6) is encoded by two independent open reading frames, *RPS6A* and *RPS6B*, which arose from genome duplication. Two phosphorylation sites (Ser232 and Ser233) exist at the C-terminal region of Rps6a and Rps6b that correspond to Ser235 and Ser236 in the human protein. Mutation of the Ser232/233 residues to alanine abolishes Rps6 phosphorylation status [10,11], suggesting that these sites are solely targets for the yeast equivalent of human S6K. Even though Sch9 was shown to phosphorylate Rps6 *in vitro* [9], there is no data on physiological pathways driving Rps6 phosphorylation *in vivo*. Interestingly, two studies performed in fission yeast showed that: a) TORC1 regulates phosphorylation of S6 in response to nutrients [12], and b) the AGC kinase Psk1, but not the Sch9 orthologs Sck1 and Sck2, is directly phosphorylated by TORC1 and functions as S6K [13].

Using a highly specific commercial antibody that recognizes the mammalian phospho-S6 sites, we have now found that nutrients rapidly induce Rps6 phosphorylation at Ser232/233 in a TORC1-dependent manner in *S*. *cerevisiae*. We also demonstrate that the AGC kinase Ypk3 is limiting for Rps6 phosphorylation, and propose Ypk3 as an S6K ortholog. Our data emphasize the significance of the phospho-S6 specific antibody as a valuable tool to study TORC1 activity.

## Materials and Methods

### Yeast and *Escherichia coli* growth conditions

Yeast cells were incubated at 30°C in YPD medium (10 g/l yeast extract, 20 g/l peptone and 20 g/l glucose) or in synthetic medium containing 20 g/l glucose and the appropriate selection requirements (SC). For nitrogen starvation, nitrogen-free (-N) medium (1.7 g Yeast Nitrogen base without amino acids and without ammonium sulfate and 20 g/l glucose) was used. The composition of YMM medium containing only one source of nitrogen at a final concentration of 0.5 g/l was previously described [14]. *E*. *coli* DH5α cells were used as plasmid DNA host and were grown at 37°C in LB broth supplemented with 50 μg/ml ampicillin when required. Yeast and bacterial cells were transformed using standard methods.

### Gene disruptions and plasmid construction

The relevant genotypes of the strains described in this work are shown in Table 1. If not otherwise stated, single *KanMX* deletion mutants in the BY4741 or BY4742 background were obtained from EUROSCARF. The *rps6* ^*S232A S233A*^ strain in the BY4741 background was generated by integrating a linear PCR fragment (obtained by PCR using primers 5’-CCAAGGATAAAGTGGTTAATGCTTATTCGTC-3’ and 5’-GAAAGGAGTTTTTGAACAAAAGAATTTATCTTTTACTGCTTTCTAACTTTAAGCCTTCAA AGCAGCAGCTCTTC-3’) containing the mutant form of *RPS6* (*rps6* ^*S232A S233A*^) into BY4741 *rps6AΔ* strain carrying *URA3* at the genomic locus of *RPS6A* (*rps6A*::*URA3*). Transformants were selected for loss of *URA3* marker by plating on standard 5-fluoroorotic acid (5-FOA) plates. Successful integration of *rps6* ^*S232A S233A*^ was confirmed by sequencing. The resulting strain (*rps6A* ^*S232A S233A*^ *RPS6B*) was then crossed to BY4742 *rps6BΔ* cells to obtain the BY4742 *rps6A* ^*S232A S233A*^ *rps6BΔ* strain by standard tetrad dissection techniques. BY4741 *rps6AΔ* strain was generated using pUG72-based method described by [15]. Point mutations were introduced by a reverse-PCR method. All constructs were sequenced to ensure the absence of undesired mutations. Plasmid pJU733 (pRS416-SCH9–3HA) was a gift from R. Loewith (Department of Molecular Biology Sciences. University of Geneva. Switzerland). Plasmids YEp352-PRS6KA2, YEp352-PRS6KA3, YEp352-RPS6KB1 and YEp352-RPS6KB2 plasmids were a gift from H. Takematsu (Department of biological chemistry, Kyoto University, Japan) [16].

**Table 1:** Yeast strains used in this work.

Name	Relevant genotype	Source/Reference
TB50a	MATa *trp1 leu2 ura3 his3 rme1*	[18]
RL206-4d	TB50a *TOR1-1 TOR2-1*	[29]
TS120-2A	TB50a *sch9*::*KanMX6*	[9]
SA234	TB50a *Ypk3-HA*::*KanMX6*	[29]
AGS105	TB50a *Ypk3* ^*S321A*^-*HA*::*KanMX6*	This work
AGS106	TB50a *Ypk3* ^*T490A*^-*HA*::*KanMX6*	This work
AGS107	TB50a *Ypk3* ^*S513A*^-*HA*::*KanMX6*	This work
YSBN9	MATα *FY3 ho*::*Ble ura3-52*	[31]
BY4741	MATa *his3Δ1 leu2Δ0 met15Δ0 ura3Δ0*	[32]
BY4742	MATα *his3Δ1 leu2Δ0 ura3Δ0*	[32]
*rps6a* ^*S232A S233A*^ *rps6b*Δ	BY4742 *rps6a* ^*S232A S233A*^ *rps6b*::*KanMX*	This work
DBY746	MAT*a, ura3*52 *leu2*-3112 *his*3-Δ1 *trp1*-Δ239	D. Botstein
DBY746 *sch9Δ*	DBY746 *sch9*::*URA3*	[33]
INA17-4D	MATa *ura3 leu2 his2 trp1 ade1*	[34]
INA106-3B	INA17-4D *pkh1* ^*ts*^ *pkh2*::*LEU2*	[34]

### Chemicals and antibodies

Rapamycin (LC Laboratories) was dissolved in dimethyl sulfoxide (DMSO). 2-nitro-5-thiocyanatobenzoic acid (NTCB, Sigma) was dissolved in water. 5-FOA was purchased from Thermo Scientific (R0812). Antibodies are as follows: phospho-Ser235/Ser236-S6 (#2211, Cell Signaling Technology), RPS6 (#ab40820, Abcam), S6K (#2708, Cell Signaling Technology), RSK (#9355, Cell Signaling Technology), anti-HA (#2367, Cell Signaling Technology), actin (#MAB1501, Millipore), peroxidase-Goat Anti-Mouse IgG (#115–035–174, Jackson ImmunoResearch Laboratories), and peroxidase-Monoclonal Mouse Anti-Rabbit IgG (#211–032–171, Jackson ImmunoResearch Laboratories).

### Immunoblot and chemical fragmentation analysis

Whole yeast cell lysates were obtained by resuspending the cells in lysis buffer (50 mM Tris-HCl pH 7.5, 150 mM NaCl, 15% glycerol, 0.5% Tween-20, phosphatase inhibitor mixture (PPi; 10 mM NaF, 10 mM NaN_3_, 10 mM *p*-nitrophenyl phosphate, 10 mM sodium pyrophosphate, and 10 mM β-glycerophosphate), 1 mM phenylmethylsulfonyl fluoride (PMSF), and EDTA-free protease inhibitor cocktail (Roche). One volume of glass beads was added, and cells were broken by vigorous shaking in a Fastprep (5 times for 45 s each at setting 5.5, with intervals of 3 min on ice) at 4°C. Unbroken cells and debris were removed by centrifugation at 500 ×*g* for 3 min. The protein concentration of the cleared lysate was determined by Bradford. 10 μg of protein were fractionated by SDS-PAGE in 10% polyacrylamide gels and transferred to Protran BA85 nitrocellulose membranes (GE Healthcare). Membranes were blocked with 5% BSA for 1 h at 24°C, and incubated for 1 h at 24°C or overnight at 4°C with the respective antibodies followed by the secondary antibodies at 1:10000 dilution. Immunocomplexes were visualized using Pierce ECL Western blotting substrate (Thermo Scientific). Chemiluminescence was detected using CL-Xposure films (Thermo Scientific). Preparation of cell extracts for detection of Sch9 phosphorylation was performed as described previously [14]. Cell extracts were subjected to chemical cleavage with NTCB [9].

### Growth tests and cell size determination

Yeast cell growth was monitored by measuring OD_600_ using an Ultraspec 2100 pro spectrophotometer (Amersham Biosciences). For the spot assays, four serial 1:5 dilutions starting at OD_600_ of 0.5 were spotted onto the indicated plates. Yeast cell size was quantified using a BD FACSCanto II analyzer (BD Biosciences).

All experiments were repeated at least two times with similar results and a representative experiment is shown. Statistical analysis was performed by using two-way analysis of variance (ANOVA). A p-value less than 0.05 was considered statistically significant.

## Results

### TORC1 stimulates Rps6 phosphorylation at Ser232/233

**Fig 1:**
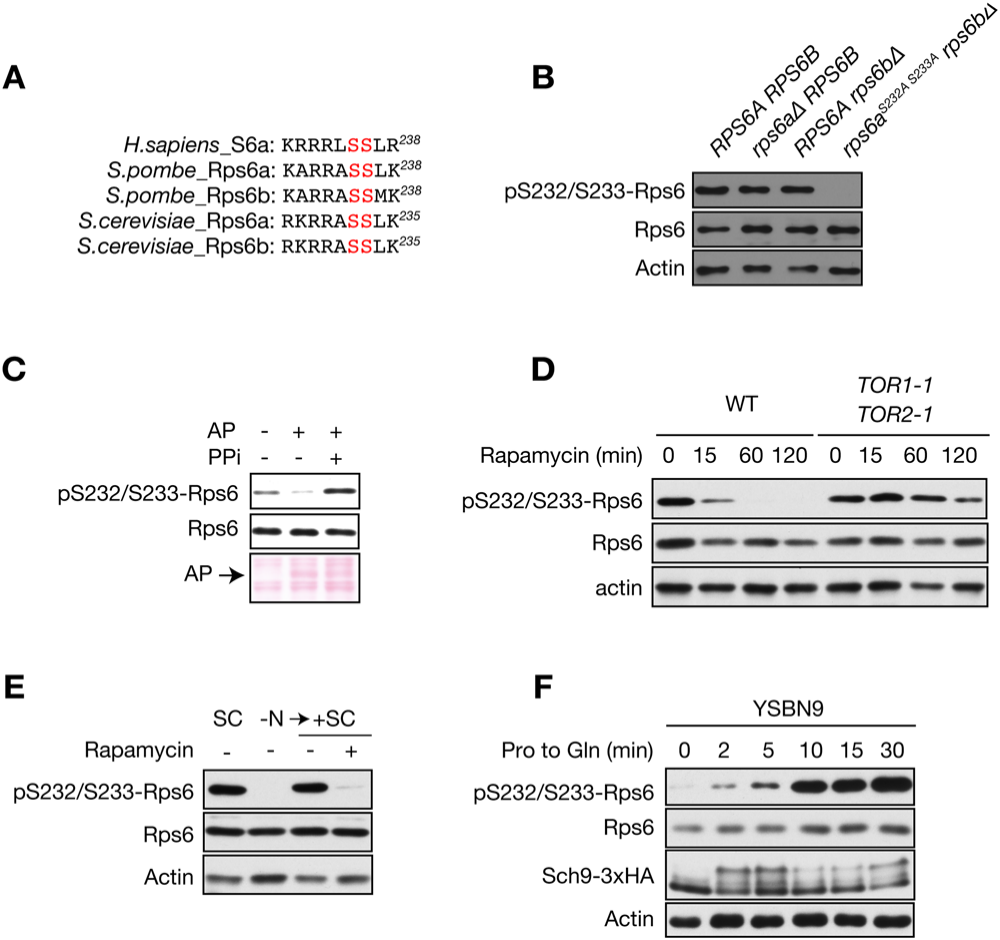
Characterization of the phospho-specific Rps6 antibody. A) C-terminal peptide sequence of human S6 and its orthologs in *S*. *pombe* and *S*. *cerevisiae*. The residues phosphorylated by S6K are highlighted in red. B) Immunoblot analysis of *S*. *cerevisiae* Rps6 phosphorylation in several *rps6* mutants in the BY4742 background. Samples from exponentially growing cultures of the indicated strains were taken. Total lysates were resolved by SDS—PAGE on 10% polyacrylamide gels and analyzed by immunoblot. Actin was used as loading control. C) Samples of exponentially growing TB50a cells were taken. Cells were lysed in lysis buffer either in the absence (lanes 1 and 2) or presence (lane 3) of phosphatase inhibitor mixture (PPi). Total protein lysates were incubated with 2 U of alkaline phosphatase (Roche) for 30 min at 37°C and analyzed by immunoblot as in B. Ponceau S staining of the membrane indicates the presence of alkaline phosphatase. D) Immunoblot analysis of Rps6 phosphorylation in WT and rapamycin-resistant *TOR1–1 TOR2–1* cells. Cells were treated with rapamycin (200 ng/ml) for the indicated times. Total lysates were analyzed as in B. E) Immunoblot analysis of *S*. *cerevisiae* Rps6 phosphorylation in WT cells. Exponentially growing cells in SC medium were shifted to nitrogen-free medium and cultured for 1 h. Then, SC medium containing 200 ng/ml rapamycin or drug vehicle was added and growth was resumed for 1 h. Total lysates were analyzed as in B. F) Immunoblot analysis of Sch9 and Rps6 phosphorylation upon glutamine stimulation. YSBN9 cells expressing Sch9–3xHA were grown in YMM with proline as the only nitrogen source. Then, glutamine (final concentration of 0.5 g/l) was added and samples were taken at the indicated times. To assess the Sch9 C-terminal phosphorylation state, protein samples were chemically cleaved with NTCB.

TORC1-mediated phosphorylation sites in S6 are conserved from yeast to human. In *S*. *cerevisiae*, Ser232 and Ser233 correspond to Ser235 and Ser236 in human S6 (Fig. 1A). S6 phosphorylation is widely used to monitor mTORC1 activity. Using a highly specific commercial anti-phospho-S6 (Ser235/236) antibody, we tested whether we could detect Rps6 phosphorylation in *S*. *cerevisiae*. Under nutrient rich conditions, we detected a 27 kDa protein that corresponds in size to Rps6 (Fig. 1B). To demonstrate that the antibody indeed recognizes only phosphorylated Rps6, we tested the phospho-specific antibody using *rps6* mutants. Single deletion of *RPS6A* or *RPS6B* did not affect Rps6 phosphorylation. Serine-to-alanine mutation of the two serine residues in Rps6a combined with *RPS6B* deletion (*rps6a* ^*S232A S233A*^ *rps6bΔ*) abolished Rps6 phosphorylation (Fig. 1B). Moreover, treatment of protein lysates with alkaline phosphatase reduced Rps6 phosphorylation (Fig. 1C). These findings confirm that Rps6 phosphorylation sites are present in *S*. *cerevisiae* and validate the specificity of the anti-phospho-S6 antibody. We next asked whether Rps6 phosphorylation is regulated by TORC1. Inhibition of TORC1 by rapamycin treatment decreased Rps6 phosphorylation in wild-type (WT) cells but failed to decrease Rps6 phosphorylation in a rapamycin-resistant *TOR1–1 TOR2–1* strain (Fig. 1D). Since TORC1 activity is controlled by nutrients, in particular by nitrogen source availability [14,17,18], we next investigated if Rps6 phosphorylation is also regulated by the nitrogen source. Nitrogen starvation resulted in decreased Rps6 phosphorylation, and re-stimulation of nitrogen-starved cells increased Rps6 phosphorylation (Fig. 1E). Thus, Rps6 phosphorylation is regulated by TORC1. We recently demonstrated that TORC1 activity is stimulated by glutamine using the phosphorylation state of Sch9 as a readout [14]. Thus, we next compared the kinetics of Rps6 and Sch9 phosphorylation in cells grown in the presence of the less-preferred nitrogen source proline upon stimulation with glutamine. Both Sch9 and Rps6 phosphorylation increased within 2 minutes after the addition of glutamine (Fig. 1F). After this initial increase, Sch9 phosphorylation decreased at 10 min to rise again at 30 min. Rps6 phosphorylation increased until 10 min after glutamine addition and then remained constant (Fig. 1F). Overall, these results underscore the validity of Rps6 phosphorylation as a specific readout for TORC1 activity *in vivo*.

### Ypk3, but not Sch9, is required for nutrient-stimulated Rps6 phosphorylation

**Fig 2:**
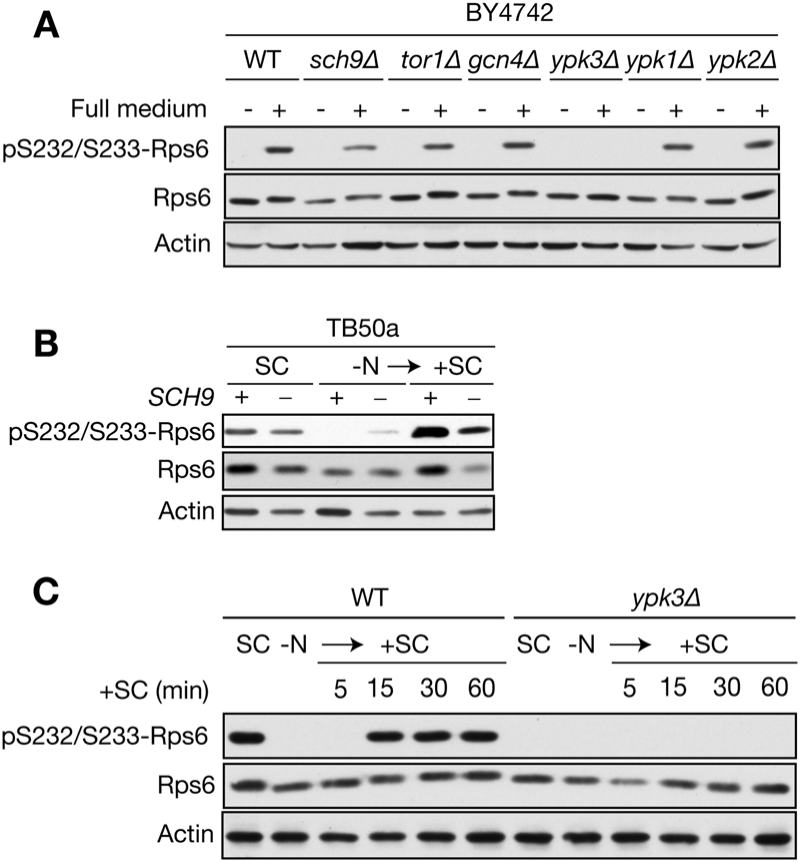
Ypk3, but not Sch9, is required for Rps6 phosphorylation at Ser232/233. A) The indicated strains in the BY4742 background were grown in synthetic complete medium for 30 h (stationary phase) and then shifted to fresh medium (+) for 1 h. Total lysates were analyzed by Immunoblot using anti-phospho-S6 antibody. B) Immunoblot analysis of Rps6 phosphorylation in WT and Sch9-deficient cells in the TB50a background. Cells were shifted from nitrogen-free medium to SC medium as in Fig. 1E and aliquots were taken at the indicated time points. C) Immunoblot analysis of Rps6 phosphorylation in WT and *ypk3Δ* cells. Cells were shifted from nitrogen-free medium to SC medium as in Fig. 1E and aliquots were taken at the indicated time points.

We next examined the role of Sch9 in Rps6 phosphorylation *in vivo*. Sch9 is a downstream target of TORC1 and was previously been shown to phosphorylate Rps6 *in vitro*. Unexpectedly, *sch9Δ* cells, similar to WT cells, were able to phosphorylate Rps6 after shifting cells from deplete medium (stationary phase cells) into fresh, complete medium (Fig. 2A). To identify the kinase that is requisite for Rps6 phosphorylation, we screened several mutants known to be involved in TORC1 signaling or that resemble features of the human S6K. We identified Ypk3, an AGC kinase that is homologous to the TORC2-targeted kinases Ypk1 and Ypk2 [19], as a promising candidate. Cells lacking Ypk3, but not Ypk1 or Ypk2, were defective for Rps6 phosphorylation under nutrient-rich conditions (Fig. 2A). We further validated, in a different genetic background, our results demonstrating that Sch9 plays little-to-no role in Rps6 phosphorylation. Rps6 phosphorylation upon stimulation of nitrogen-starved *sch9Δ* cells with fresh medium was comparable to that observed in WT cells (Fig. 2B). However, cells lacking Sch9 expressed lower amounts of Rps6 protein (Fig. 2B), an observation that is consistent with the role of Sch9 in promoting ribosomal protein gene expression [9,20]. Next, we examined the kinetics of Rps6 phosphorylation upon stimulation of nitrogen-starved WT or *ypk3Δ* cells with fresh medium. Rps6 phosphorylation in WT cells increased during the 15 min after stimulation and thereafter remained constant up to 60 min, whereas in *ypk3Δ* cells Rps6 phosphorylation was completely absent (Fig. 2C). Together our data demonstrate that Ypk3 is rate-limiting for phosphorylation of Rps6 at Ser232/233.

### TORC1 and Pkh1/2 kinases are essential for Ypk3 activity toward Rps6

**Fig 3:**
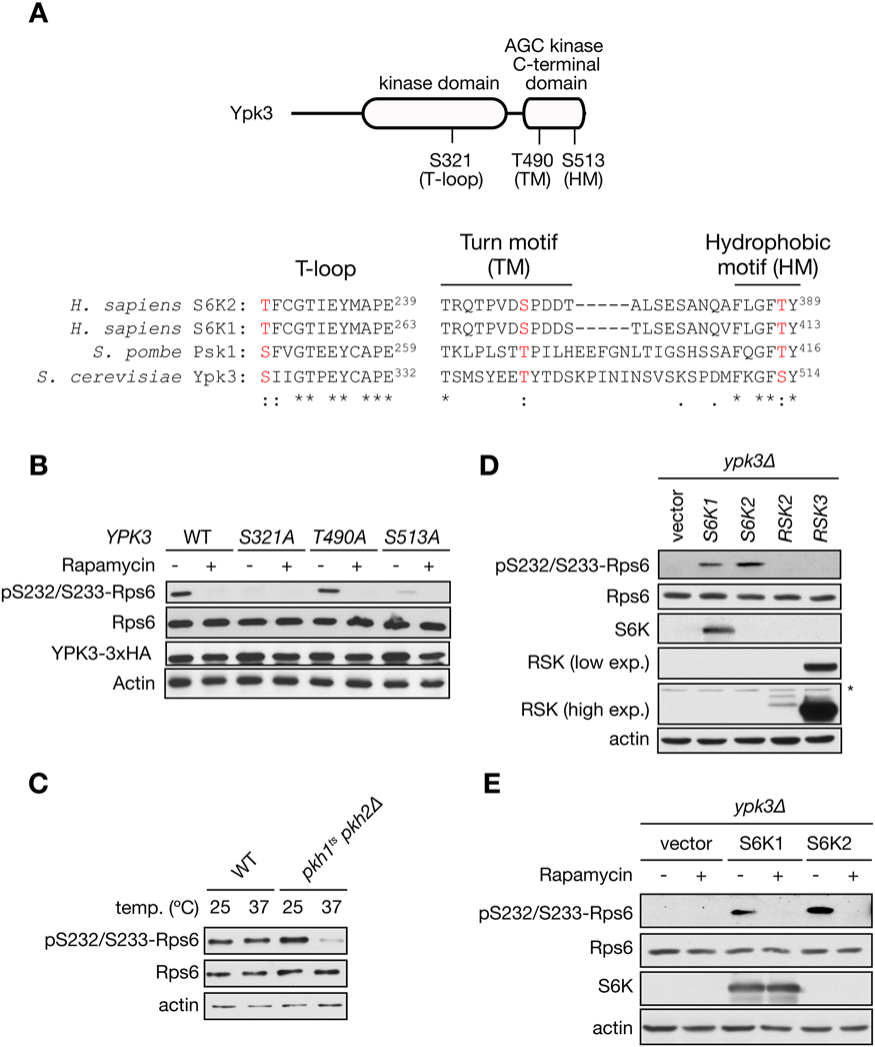
Ypk3 functions as a Rps6 kinase *in vivo*. A) Top: Schematic depiction of Ypk3 showing the phosphorylation sites in the T-loop, turn motif (TM) and hydrophobic motif (HM); bottom side: conserved amino acids (phosphorylation sites) within the sequence alignment of human S6K and *S*. *pombe* and *S*. *cerevisiae* counterparts are highlighted in red. An asterisk (*) indicates positions which have a fully conserved residue. Colon (:) and period (.) indicate conservation between residues of strongly and weakly similar properties, respectively. B) Immunoblot analysis of Rps6 phosphorylation in phosphorylation-deficient Ypk3 mutants. Cells expressing Ypk3-HA or the phosphorylation-deficient mutants were treated with rapamycin (200 ng/ml) for 1 h. Total lysates were analyzed by inmmunoblot. C) Analysis of Rps6 phosphorylation status in cells lacking Pkh1/2. Exponentially growing cells of the strains INA17–4D (WT) and INA106–3B (*pkh1* ^*ts*^ *pkh2Δ*) at 25°C were shifted to 37°C for 1 h. D) Immunoblot analysis of Rps6 phosphorylation in *ypk3Δ* cells transformed with the indicated plasmids containing the human S6K1 and S6K2 as well as other AGC family members including RSK2 and RSK3 under the constitutive *ADH1* promoter. The asterisk indicates the presence of a non-specific band. E) Rps6 phosphorylation in *ypk3Δ* cells transformed with the indicated plasmids. Cells were treated with rapamycin (200 ng/ml) for 1 h.

To be active, mammalian S6K requires phosphorylation at three important regulatory motifs: the T- or activation loop (T-loop) which is located in the catalytic domain, the hydrophobic motif (HM) which is located in a non-catalytic region following the kinase domain, and the turn motif (TM), another phosphorylation site that promotes S6K integrity. mTORC1 phosphorylates S6K on the HM and TM whereas PDK1 phosphorylates S6K on the T-loop [21,22]. All three regulatory motifs are present in Ypk3 (Fig. 3A). Interestingly, using a mass-spectrometry-based proteomics approach, it was shown recently that Ypk3 is phosphorylated *in vivo* at Ser513 (HM) in a TORC1-dependent manner [23]. To investigate whether phosphorylation of the motifs in Ypk3 is important for Ypk3 kinase activity toward Rps6, we generated phosphorylation-deficient mutants by converting the Ser/Thr residues to alanine. In line with mammalian and *S*. *pombe* S6K, mutation of Ser321 (T-loop) or Ser513 to alanine abolished Rps6 phosphorylation under normal growth conditions, while mutation of Thr490 in the TM had no impact on Rps6 phosphorylation (Fig. 3B). Altogether, these data suggest that Ypk3 activation requires T-loop and HM phosphorylation to maintain signaling to Rps6. Since Ser321 in the T-loop is required for Rps6 phosphorylation, we sought to investigate if this effect is mediated by the PDK1 orthologs Pkh1 and Pkh2 [24]. Because cells lacking both Pkh1 and Pkh2 are inviable, we assessed phosphorylation of Rps6 in a conditional *pkh1Δ pkh2* ^*ts*^ mutant. Rps6 phosphorylation was completely abolished in cells shifted to non-permissive temperature (37°C) for 1 h (Fig. 3C). These results indicate that similar to PDK1 in mammals, yeast PDK1 orthologs are required for Rps6 phosphorylation. To further confirm that Ypk3 is equivalent to mammalian S6 kinase, we expressed human S6K1 and S6K2 isoforms as well as other AGC kinase family members including ribosomal protein S6 kinase alpha-3 and 2 (RSK2 and RSK3) under the constitutive *ADH1* promoter in cells lacking Ypk3. Heterologous expression of human S6K1 or S6K2 was sufficient to restore Rps6 phosphorylation in *ypk3Δ* cells (Fig. 3D). In contrast, heterologous expression of RSK2 and RSK3 did not restore Rps6 phosphorylation (Fig. 3D). Interestingly, human S6K-mediated phosphorylation of Rps6 was also sensitive to rapamycin (Fig. 3E), indicating a functional signal transduction pathway. Taken together, our findings demonstrate that Ypk3 is a critical component of the TORC1 pathway and that loss of Ypk3 can be complemented by human S6K.

### Mutants defective for Rps6 phosphorylation do not exhibit growth defects

**Fig 4:**
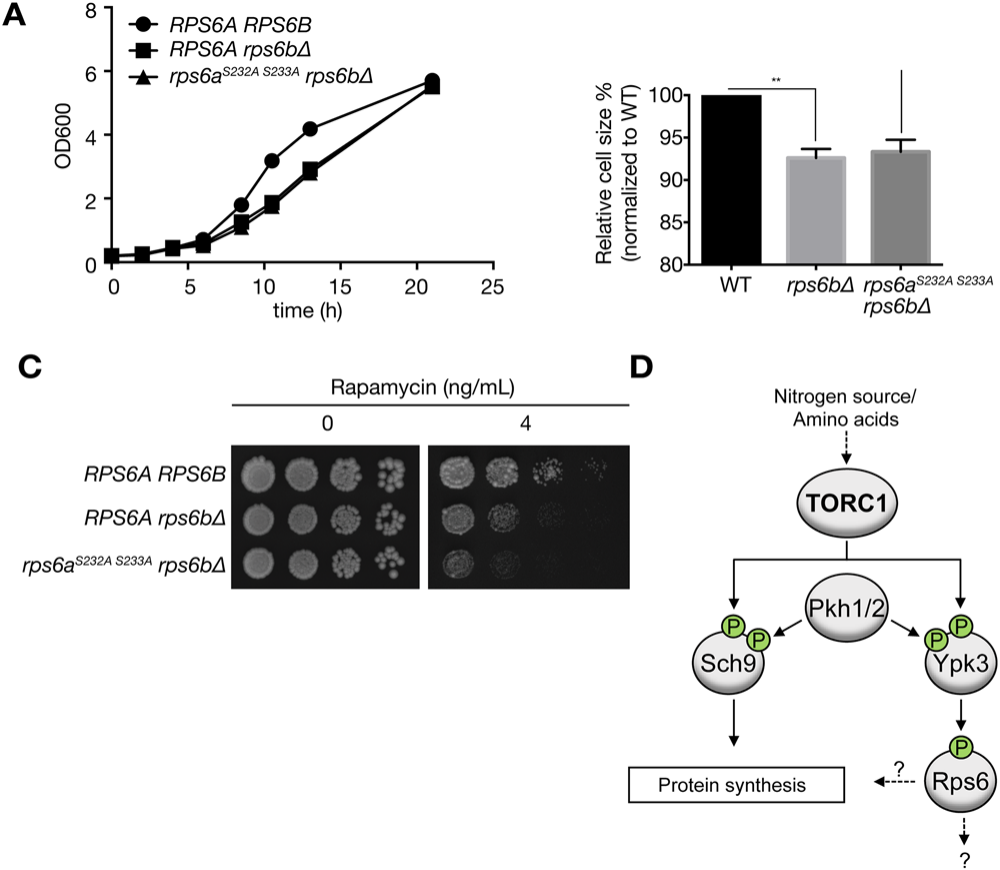
Phenotypic characterization of the Rps6 phosphorylation deficiency. A) WT strain BY4742 and the indicated mutants were tested for growth in YPD medium. Growth was monitored at the indicated times. Data are presented as the mean ± S.E.M. of triplicate determinations and correspond to a representative experiment among three. Statistical analysis was performed by using two-way ANOVA. WT *vs*. *rp6bΔ* (significant: p < 0.001), WT vs. *rps6a* ^*S232A S233A*^ *rp6bΔ* (significant: p = 0.001), *rp6bΔ v*s. *rps6a* ^*S232A S233A*^ *rp6bΔ* (not significant: p = 0.153). B) Cell size of the indicated strains was measured. Two-way ANOVA analysis was performed. **, p<0.01; ns, not significant. C) WT strain BY4742 and the indicated mutants were spotted onto YPD plates containing either 4 ng/ml of rapamycin or drug vehicle alone. Plates were grown for 3 days at 30°C. Pictures correspond to a representative experiment among three. D) Model of regulation of Rps6 phosphorylation by TORC1-Ypk3.

The biological role of S6 phosphorylation in yeast is not well understood. Here we compared cell proliferation, cell size and rapamycin sensitivity of WT, *rps6bΔ*, and phosphorylation-deficient *rps6a* ^*S232A S233A*^ *rps6bΔ* cells. In agreement with previous observations [10,11,25], *rps6bΔ* cells proliferated slower than WT cells, indicating that Rps6b is important for growth. However, the phosphorylation-deficient mutant resembled *rps6bΔ* cells (Fig. 4A). Cell size was reduced by 6% in *rps6bΔ* mutants and *rps6a* ^*S232A S233A*^ *rps6bΔ* cells showed a similar decrease in size (Fig. 4B). When treated with rapamycin, *rps6a* ^*S232A S233A*^ *rps6bΔ* cells exhibited additional mild hypersensitivity compared to *rps6bΔ* cells (Fig. 4B). Thus, our data indicate that Rps6a phosphorylation is not important for cellular proliferation in *rps6bΔ* cells.

## Discussion

### Sch9 *vs*. Ypk3 as a S6K in *S*. *cerevisiae*

In budding yeast, S6K function was previously ascribed to Sch9 which phosphorylates Rps6 at Ser232 and Ser233 *in vitro* [9]. However, there is no experimental evidence that Sch9 is required for Rps6 phosphorylation *in vivo*. Using a highly specific commercial phospho-Rps6 antibody, our study for the first time demonstrates that TORC1 stimulates Rps6 phosphorylation independently of Sch9. Our findings further indicate that Ypk3 functions as S6K in *S*. *cerevisiae* downstream of TORC1. These results are consistent with a previous study which, based on sequence homology, suggested that Ypk3 could be a S6K ortholog [26]. Consistent with mammalian and *S*. *pombe* S6K orthologs, phosphorylation in T-loop and HM motif is indispensable for Ypk3 kinase activity towards Rps6. Moreover, loss of *YPK3* was complemented by heterologous expression of human S6K1 and S6K2 (Fig. 3D) and even more importantly, human S6K-mediated phosphorylation of Rps6 was controlled by *S*. *cerevisiae* TORC1 in a rapamycin-sensitive manner (Fig. 3E). Taken together, we propose Ypk3 as the *bona fide* S6 kinase in *S*. *cerevisiae*. Further work is required to characterize in detail how Ypk3 mediates downstream signals of TORC1.

### Phospho-Rps6 antibody is a useful tool to study TORC1 signaling

Measurement of TORC1 activity in *S*. *cerevisiae* has relied on chemical cleavage of Sch9 and mobility shift in SDS-PAGE, or non-commercial phospho-specific antibody against Sch9 [9]. In this study we show that a commercially available antibody generated against phosphorylated human S6 is able to detect endogenous Rps6 phosphorylation in *S*. *cerevisiae*. Importantly, the Rps6 phosphorylation is positively regulated by nutrients in a TORC1-dependent manner. Thus, Rps6 phosphorylation is a valuable readout to study TORC1 signaling in *S*. *cerevisiae*.

### Role of PKA in Ypk3 phosphorylation

Ypk3 is phosphorylated by PKA *in vitro* [27,28] and *in vivo* at Thr82 and Ser90 [29]. This *in vivo* phosphorylation is rapamycin sensitive, suggesting that TORC1 is an upstream regulator of PKA toward Ypk3. The functional significance of Ypk3 phosphorylation through PKA is unclear, but the phosphorylation sites are not conserved in *S*. *pombe* or human S6K. However, our results indicate that rapamycin decreases Rps6 phosphorylation in *bcy1Δ* cells in which PKA is constitutively active (data not shown). This suggests that TORC1 controls Rps6 phosphorylation via Ypk3 but independently of PKA. Further experiments are required to elucidate the crosstalk of TORC1 and PKA towards Ypk3 activity.

### The role of S6K and S6 phosphorylation in yeast and mammals

What is the functional relevance of Rps6 phosphorylation? The biological function of Rps6 phosphorylation in yeast is obscure. The first attempts to elucidate the physiological role of Rps6 phosphorylation were carried out in *S*. *cerevisiae*. Mutation of Ser232/233 to alanine in Rps6 had no detectable effect on growth on different carbon sources, sporulation, or sensitivity to heat shock [10,11]. In agreement with these observations, we failed to detect a defect in proliferation and cell size in Rps6 phosphorylation-deficient cells. Consistently, *ypk3Δ* mutants as well as *ypk3* ^*S321A*^ and *ypk3* ^*S513A*^ cells proliferated like WT cells (data not shown). Knock-in mice in which the five S6K target serine residues in S6 are mutated to alanine (*S6* ^*P−/−*^ mice) are viable, fertile, and show no detectable differences in body size. Global translation is not impaired, but rather enhanced, in *S6* ^P−/−^ MEFs and liver [7,30], which is consistent with the fact that *S6K1* ^−/−^ *S6K2* ^−/−^ cells maintain global translation [7]. It is tempting to speculate that the absence of Rps6 phosphorylation activates a compensatory mechanism through which global protein translation is maintained. Indeed, liver-specific *S6K1* ^*−/−*^ *S6K2* ^*−/−*^ and *S6* ^*P−/−*^ mice showed more phosphorylation and inhibition of 4E-BP, leading to the release of 4E-BP from eIF4E and thus upregulating protein synthesis [7]. Identification of such compensatory mechanism in *S*. *cerevisiae*, if any, will shed light on the role of Rps6 phosphorylation in cell growth and proliferation.
